# Antioxidant and Anti-Proliferative Activity of Essential Oil and Main Components from Leaves of *Aloysia polystachya* Harvested in Central Chile

**DOI:** 10.3390/molecules26010131

**Published:** 2020-12-30

**Authors:** Alejandra Catalina Moller, Carol Parra, Bastian Said, Enrique Werner, Susana Flores, Joan Villena, Alessandra Russo, Nelson Caro, Iván Montenegro, Alejandro Madrid

**Affiliations:** 1 Escuela de Tecnología Médica, Facultad de Medicina, Universidad de Valparaíso, Angamos 655, Reñaca, Viña del Mar 2520000, Chile; alejandra.moller@uv.cl; 2 Laboratorio de Investigación en Nutrición y Alimentos (LINA), Departamento Disciplinario de Nutrición, Facultad de Ciencias de la Salud, Universidad de Playa Ancha, Valparaíso CP 2340000, Chile; carol.parra@upla.cl; 3 Departamento de Química, Universidad Técnica Federico Santa María, Av. Santa María 6400, Vitacura, Santiago 7630000, Chile; bastian.said@usm.cl; 4 Departamento de Ciencias Básicas, Campus Fernando May, Universidad del Bío-Bío. Avda. Andrés Bello 720, casilla 447, Chillán 3780000, Chile; ewerner@ubiobio.cl; 5 Laboratorio de Productos Naturales y Síntesis Orgánica (LPNSO), Departamento de Química, Facultad de Ciencias Naturales y Exactas, Universidad de Playa Ancha, Avda. Leopoldo Carvallo 270, Playa Ancha, Valparaíso 2340000, Chile; s.flores.gonzalez@gmail.com; 6 Centro de Investigaciones Biomedicas (CIB), Facultad de Medicina, Campus de la Salud, Universidad de Valparaíso, Angamos 655, Reñaca, Viña del Mar 2520000, Chile; juan.villena@uv.cl; 7 Department of Drug Sciences, University of Catania, Via S. Sofia 64, 95125 Catania, Italy; alrusso@unict.it; 8 Centro de Investigación Australbiotech, Universidad Santo Tomás, Avda. Ejército 146, Santiago 8320000, Chile; ncaro@australbiotech.cl; 9 Escuela de Obstetricia y Puericultura, Facultad de medicina, Universidad de Valparaíso, Angamos 655, Reñaca, Viña del Mar 2520000, Chile

**Keywords:** *Aloysia polystachya*, terpenoids, cytotoxic activity, selectivity index

## Abstract

The aim of this study was to determine, first, the chemical composition of *Aloysia polystachya* (Griseb) Moldenke essential oil, from leaves harvested in central Chile; and second, its antioxidant and cytotoxic activity. Eight compounds were identified via gas chromatography–mass spectrometry (GC–MS) analyses, with the most representative being *R*-carvone (91.03%), *R*-limonene (4.10%), and dihydrocarvone (1.07%). For *Aloysia polystachya* essential oil, antioxidant assays (2,2-diphenyl-1-picrylhydrazyl (DPPH), H_2_O_2_, ferric reducing antioxidant power (FRAP), and total reactive antioxidant potential (TRAP)) showed good antioxidant activity compared to commercial antioxidant controls; and anti-proliferative assays against three human cancer cell lines (colon, HT-29; prostate, PC-3; and breast, MCF-7) determined an IC_50_ of 5.85, 6.74, and 9.53 µg/mL, and selectivity indices of 4.75, 4.12, and 2.92 for HT-29, PC-3, and MCF-7, respectively. We also report on assays with CCD 841 CoN (colon epithelial). Overall, results from this study may represent, in the near future, developments for natural-based cancer treatments.

## 1. Introduction

The family Verbenaceae, which is comprised of some 50 species native to the American continent, distributed mainly in temperate climates and some subtropical and desert climates [1], includes *Aloysia* spp., a genus of flowering plants. Of these, *Aloysia polystachya* (Griseb) Moldenke (Verbenaceae) is an aromatic shrub that grows throughout South America and whose leaves enjoy widespread use to aromatize “*mate*” or “*tereré*” [2] (popularly known as “*Té del Burrito*” [3], a denomination originating in the Argentinean mountains), or as folk medicine to treat nausea, vomiting, dyspepsia, gastritis, and anxiety disorders [4]. Data from the literature show that alcoholic extracts of this plant exhibit biological effects, including antioxidant [5], antitumor [6], antispasmodic [7], anxiolytic [8], and antidepressant properties [9].

Nevertheless, there are few reports in the literature concerning the biological properties of essential oil from this plant. There are four reports from Argentina and one from Brazil where the chemical composition of the oils has been determined. Of the plants that grow in Argentina, the main constituents of the oils of the leaves of the species collected in the province of Cordova have been described as the monoterpenes α-tujone (83.56%), sabinene (4.61%), and limonene (1.62%) [10,11], while in species collected in the cities of Buenos Aires and Corrientes the predominant monoterpenes are carvone (83.5–84.4%), limonene (14.2–16.5%), and verbenone (1.58%) [12,13]. The essential oil from *A. polystachya* leaves grown in the southeast region of Brazil revealed a high content of carvone (80.71%) and limonene (14.65%) [14]. In addition, these studies validated the antimicrobial activity of *A. polystachya* oils against different human pathogens [10,11,12,13,14].

However, to the best of our knowledge, no studies have determined the chemical composition and/or biological activities of the essential oil of the leaves of *A. polystachya* growing in Chile. Thus, the aim of the present study was to determine the chemical constituents of the essential oil extracted from fresh leaves of *A. polystachya* growing in Chile and evaluate its antioxidant and cytotoxic activities.

## 2. Results and Discussion

**Figure 1:**
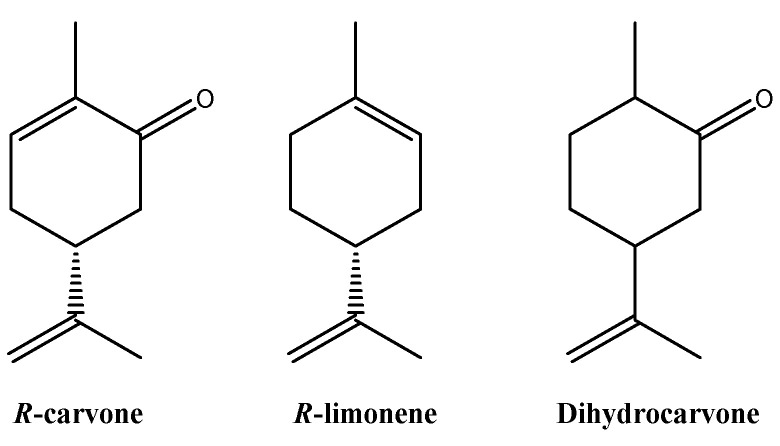
Structures of the main compounds present in EO of *A. polystachya*.

The hydrodistillation of the fresh leaves of *A. polystachya* gave light yellow oil with a yield of 1.21% (*v*/*w*). The essential oil of *A. polystachya* fresh leaves is composed mainly by oxygenated monoterpenes (93.61%), followed by hydrocarbon monoterpenes (4.10%) and hydrocarbon sesquiterpenes (1.28%) (Table 1). Eight compounds were identified in the essential oil of *A. polystachya*, which corresponded to 98.99% of the total oil analyzed, and the main components were: *R*-carvone (91.03%), *R*-limonene (4.10%), and dihydrocarvone (1.07%) (Figure 1).

Our results on *A. polystachya* essential oil from fresh leaves collected in central Chile are consistent with other studies, identifying carvone and limonene as the most abundant constituents [13]; however, our report is the first in which carvone constitutes over 90 percent of the composition. This variation in essential oil content and chemical composition is influenced by many factors, including location, plant age, climate, cultivar, distillation method, and type of distillation apparatus used [16].

The antioxidant activity of the fresh leaf essential oil of *A. polystachya*, examined using four different assays, is shown in Table 2.

While 2,2-diphenyl-1-picrylhydrazyl (DPPH) values indicate significant antioxidant activity of *A. polystachya* oil on aromatic herbal essential oils, such as rosemary (*Rosmarinus officinalis* L.), cedar (*Cedrus libani*), and lemon balm (*Melissa officinalis* L.) [17,18], it still presents low radical scavenging activity compared to reference compounds. That said, *A. polystachya* essential oil was shown to have greater redox properties than commercial standards under ferric reducing antioxidant power (FRAP) values and similar redox under total reactive antioxidant potential (TRAP) values.

The antioxidant activity of *A. polystachya* essential oil is likely due to the high oxygenated monoterpene content [19], under which carvone is most representative [20]. Carvone is an oxygenated monoterpene with a double bond conjugated with a ketone group, which gives it greater capacity to capture free radicals and better reducing power [20,21,22,23]. Indeed, high carvone content has been linked to antioxidant activity in *Mentha spicata* and *M. gracilis* essential oils [24], as well as in essential oils of different species of the Verbenaceae family like *Lippia alba* and *Phyla nodiflora* [22,25,26,27]. The antioxidant activity of *A. polystachya* essential oil is further enhanced by the presence of unsaturated terpenes, like limonene, a monocyclic monoterpene capable of inhibiting free radicals and lipid peroxidation that prevents cell damage by reducing blood pressure and cardiovascular response to stress [28,29]; and other monoterpenes with hydroxyl substitutes, such as terpineol and linalool [30,31].

The inhibitory effects of the essential oil (IC_50_ values) are presented in Table 3. The tested *A. polystachya* oil showed prominent cytotoxic activity against all cancer cell lines used in the study.

Based on criteria established by the National Cancer Institute (NCI) Plant Screening Program, a crude extract of a medicinal plant is considered to have potential if the in vitro cytotoxicity studies reported an IC_50_ value of less than 20 µg/mL following incubation of 48–72 h [32]. In the present study, the oil showed pronounced effects against HT-29 (colon), PC-3 (prostate), and MCF-7 (breast), with IC_50_ values of 5.85 ± 0.39, 6.74 ± 0.03, and 9.53 ± 0.45 µg/mL, respectively, results which are consistent with NCI guidelines. Notably, these were significantly lower than the IC_50_ value obtained for normal cell line, 27.81 ± 0.21 µg/mL. Moreover, *A. polystachya* oil cytotoxic activity was much higher than 5-fluorouracil (5-FU) in all cell lines tested, although lower than daunorubicin against breast and prostate cell lines. Nevertheless, these results—which are supported by previous in vivo assays of alcohol extracts of this plant [6]—demonstrate the anti-proliferative potential of this species, and particularly so against the colon tumor cell line. Furthermore, essential oils with IC_50_ values under 30 µg/mL are typically classified as promising anti-cancer agents [33]. This report is therefore indicative that *A. polystachya* oil may be a potential substrate for the development of new drugs against this disease.

According to recent studies [34,35,36], a selectivity index (SI) value of more than three was considered highly selective against cancer cells. In vitro activity of tested samples against the CCD 841 CoN cell line were used to calculate the selectivity indices (Equation (3)), shown in Table 3. The *A. polystachya* essential oil presented the best selectivity indices against the HT-29 and PC-3 cell lines (4.75 and 4.13 respectively), and 1.6 times less selectivity against the MCF-7 cell line. Based on these results (and selectivity criteria reported in the literature), the essential oil can be considered a selective agent for the MCF-7 cell line, at values above 2, and for PC-3 and HT-29 cells, at values greater than 3 [32,33,34,35]. Finally, the selective activity of the essential oil against the HT-29 cell line is comparable to that of 5-FU, and superior to daunorubicin.

The activity is most likely a synergistic effect among the major monoterpenes found in the *A. polystachya* essential oil [37]. Among the identified terpenes, d-limonene has a demonstrated ability to inhibit cell proliferation, e.g., by inducing apoptosis in lung, stomach, and gastric liver cells [38]. Furthermore, carvone has been shown to be cytotoxic in some tumor cell lines [39,40]; for example, *M. spicata* oil—with similar carvone content (65.33%)—showed similar cytotoxic activity against the HeLa cell line [41], with IC_50_ values below 10 µg/mL. Besides, carvone is bioactive compound that contribute to the pharmacological activity of the various essential oils in which they are found [42]. Previous studies showed that the use of carvone and limonene mixtures enhanced the cytotoxic activity of each of the monoterpenes separately [43]. In turn, reports suggest that oxygenated monoterpenes, such as terpineol and linalool present in the oil, contribute to the cytotoxic potential of vegetable oils [44,45]. In addition, hydrocarbon sesquiterpenes, such as *E*-caryophyllene and α-curcumene, would contribute to the synergistic effect of *A. polystachya* essential oil, since both compounds possess significant anticancer activities, affecting the growth and proliferation of numerous cancer cells [46,47]. However, the individual compounds did not exhibit cytotoxic activity against the tumor lines tested. Despite the fact that carvone and limonene did not show cytotoxicity in this study as in other works [20,48,49], previous studies showed cytotoxic activity against prostate and breast cancer cell lines [50,51,52]. These monoterpenes are present in low concentrations in food and have applications in industry and agriculture, increasing the human exposure to these compounds [53,54]. Finally, our data may represent, in the near future, developments for natural-based cancer treatments or a potential preservative agent.

## 3. Materials and Methods

### 3.1. General Data

All reagents, *R*-carvone, *R*-limonene, and dihydrocarvone were purchased from Sigma-Aldrich Co. (St. Louis, MO, USA), GIBCO BRL Life Technologies (Grand Island, NY, USA), and Santa Cruz Biotechnology (Santa Cruz, CA, USA).

### 3.2. Equipment

The analysis of the essential oil was carried out by gas chromatography–mass spectrometry (GC–MS) using a Hewlett-Packard GC/MS 6890 coupled to a Hewlett-Packard 5973 mass-selective detector (electron ionization, 70 eV, Palo Alto, CA, USA) and equipped with a capillary HP-5 MS column. Antioxidant assays were determined in a UV–Vis spectrophotometer (Jenway 6320D, Bibby Scientific Limited, Beacon Road, Stone, Staffordshire ST15 0SA, UK). Anti-proliferative assay was determined in a microplate reader (SpectraMax, Winooski, VT, USA).

### 3.3. Plant Material

Plant samples were collected from Villa Alemana, Valparaiso Region, Central Chile (S: 33.0497°, W: −71.3927°) at an altitude of approximately 133 m during the spring in October 2019. Botanical identification and authentication was verified by Mr. Patricio Novoa, and a voucher specimen (AP-1019) was deposited at the Natural Products and Organic Synthesis Laboratory of Universidad de Playa Ancha, Valparaíso, Chile.

### 3.4. Preparation of Essential Oil

The essential oil was extracted from fresh leaves of *A. polystachya* (500 g) ground in a knife mill by steam distillation carried out using a Clevenger-type apparatus for 4 h [55]. Thereafter, the hydrolate was subjected to liquid-liquid partition in a separatory funnel and three washes with three 10 mL portions of dichloromethane. The essential oil sample was stored at −4 °C until further chemical and biological tests.

### 3.5. Chemical Analysis

The essential oil of *A. polystachya* was analyzed by GC–MS. The working conditions were as follows: injector temperature, 250 °C; detector temperature, 280 °C; carrier gas, He at 1.25 mL/min; and oven temperature program: 35 °C for 5 min, increase to 260 °C at 5 °C/min, and then 260 °C for 5 min. Compounds in the chromatogram (see Figure S1 Supplementary Material) were identified by comparison of their mass spectra with those in the NIST 2014 library database, and by comparison of their retention index with those reported in the literature [15], for the same type of column or those of commercial standards, when available. The retention indices were determined under the same operating conditions in relation to a homologous n-alkanes series (C_8_–C_36_) by Equation (1):

RI = 100 × (n + Tr_(unknown)_ − Tr_(n)_/Tr_(N)_ − Tr_(n)_) (1)

where n = the number of carbon atoms in the smaller *n*-alkane; N = the number of carbon atoms in the larger n-alkane; and Tr = the retention time. The components’ relative concentrations were obtained by peak area normalization.

### 3.6. Antioxidant Assays

#### 3.6.1. 2,2-Diphenyl-1-picrylhydrazyl (DPPH) Radical Scavenging Activity

The DPPH free radical scavenging activity was estimated by assay based on the method described in the literature [56]. Briefly, 2.0 mL of 0.5 mmol/L DPPH in ethanol was mixed with 100 μL of essential oil of *A. polystachya* (0.001, 0.01, 0.1, and 1.0 mg/mL). After 20 min incubation, the absorbance was measured at 517 nm. Trolox and gallic acid were used as positive controls. The percentage of free radical-scavenging capacity was calculated by Equation (2):

RSC% = 100% × (A_control_ − A_sample_)/A_control_ (2)

where A_sample_ is the absorbance of DPPH mixed with essential oil and A_control_ is the absorbance of DPPH in which sample has been replaced with ethanol. All measurements were performed in triplicate and reported as the average value. The IC_50_ value was determined by linear regression analysis from the obtained radical scavenging capacity (RSC) values.

#### 3.6.2. H_2_O_2_ Scavenging Activity

The H_2_O_2_ scavenging activity was determined according to a previously described method [57]. A solution of hydrogen peroxide (40 mM) was prepared in phosphate buffer (pH 7.4). The samples (from 2.5 to 10 μL of the 0.1% essential oil) were added to a hydrogen peroxide solution (0.6 mL, 40 mM). Absorbance of hydrogen peroxide at 230 nm was determined after 10 min. Butylated hydroxytoluene (BHT) and Trolox were used as positive controls. The percentage of scavenging of hydrogen peroxide by the essential oil was calculated using Equation (3):

H_2_O_2_% = 100% × (A_0_ − A_1_)/A_0_ (3)

where A_0_ represents the absorbance of the control and A_1_ represents the absorbance in the presence of the essential oil and standards. IC_50_ was the effective concentration at which 50% of hydrogen peroxide was scavenged.

#### 3.6.3. Ferric Reducing Antioxidant Power (FRAP) Assay

FRAP assay was carried out using the above method [58]. FRAP test solution was prepared using FeCl_3_·6H_2_O in distilled water (final concentration of Fe(III) in the solution was 20 mM), 2,4,6-tri(2-pyridyl)-s-triazine (TPTZ) in 40 mM HCl (final concentration of TPTZ was 10 mM), and 0.3 M CH_3_COOH/CH_3_COONa buffer solution at pH = 3.6. The FRAP reagent was prepared daily as follows: acetic acid buffer, TPTZ solution, and FRAP test solution were mixed in this order at the volume ratio of 10:1:1. A 3.0 mL aliquot of FRAP reagent was mixed with 300 μL of deionized water and 100 µL of methanolic essential oil solution (1.0 mg/mL). The mixture was vigorously shaken for 30 s and left in the dark at 37 °C for 30 min. Subsequently the absorbance was measured at 593 nm using ethanol as the blank solution. The obtained absorbance values were interpolated in a Trolox calibrate curve (0–200 mg/L) and the FRAP values were expressed in mM Trolox equivalent antioxidant capacity (mM TEAC). BHT and gallic acid were used as positive controls. All of the measurements were performed in triplicate.

#### 3.6.4. The Total Reactive Antioxidant Potential (TRAP) Assay

The TRAP of the essential oil was determined by ABTS^+^ (2,2′-azinobi(3-ethylbenzothiazoline-6-sulfonic acid)) assay [57]. The ABTS^+^ (2,2′-azinobi(3-ethylbenzothiazoline-6-sulfonic acid)) radical solution (150 µM) was mixed with 2,2′-azo-bis(2-amidino propane) (ABAP) solution (10 mM) in phosphate-buffered saline (PBS), pH 7.4 solution (100 mM). The mixture was incubated at 45 °C for 30 min. 10 µL of sample solution was added to 990 µL of the resulting blue-green ABTS radical solution. The decrease of absorbance of TRAP solutions and ABTS as blank were recorded after 30 s at room temperature. Then, the absorbance of the samples was measured at 734 nm. The total antioxidant capacity (TRAP) of the essential oil was expressed in mM Trolox equivalents (TEAC), using a standard curve of Trolox (0–120 mg/L). BHT and gallic acid were used as positive controls. All measurements were replicated three times.

### 3.7. Cell Lines and Culture Conditions

In this study, we used three different tumor cell lines: human breast adenocarcinoma (MCF-7), human prostate adenocarcinoma (PC-3), and human colorectal adenocarcinoma (HT-29). A normal human cell line (colon epithelials, CCD 841 CoN) was included to evaluate the possible selective activity of the essential oil. The different cell lines were maintained as monolayers in a plastic culture medium (HAM-F10+DMEM, 1:1) supplemented with 10% fetal bovine serum, as well as antibiotics (0.01 mg/mL streptomycin and 0.005 mg/mL penicillin). The cells were incubated at 37 °C in a humidified 5% CO_2_ atmosphere.

### 3.8. Anti-Proliferative Assay

The panel of cancer cells were seeded at a density of 2 × 10^4^ cells/well into 96-well plates and assayed as described previously [59]. Test compounds were solubilized just prior to the experiment in 0.1% DMSO. Briefly, cells were treated with increasing concentrations of essential oil (0.625–100 µg/mL) for 72 h at 37 °C in 5% CO_2_. The cells which received only the medium containing 0.1% DMSO served as the control group. At the end of essential oil exposure, cells were fixed with 50% trichloroacetic acid at 4 °C (TCA final concentration 10%). After washing with distilled water, cells were stained with 0.1% sulforhodamine B (Sigma-Aldrich, St. Louis, MO, USA), dissolved in 1% acetic acid (50 µL/well) for 30 min, and subsequently washed with 1% acetic acid to remove unbound stain. Protein-bound stain was solubilized with 100 µL of 10 mM unbuffered Tris base. The cell density was determined using a fluorescence plate reader (wavelength 540 nm). Daunorubicin and 5-fluorouracil (5-FU) were used as positive controls. Values shown are the mean + SD of three independent experiments in triplicate. Finally, Sigma Plot software (Systat Software, San Jose, CA, USA) was used to calculate the IC_50_ value.

### 3.9. Selective Index

The selectivity index (SI) is the ratio between the IC_50_ value of the *A. polystachya* essential oil obtained for CCD 841 CoN cells and the value found for the cancer cell line (Equation (4)).

SI = IC_50 (CCD 841 CoN)_/IC_50(cancer cell)_ (4)

where a SI > 3 was considered to belong to a selective sample [33,34,35,36].

### 3.10. Statistical Analysis

The data were reported as the mean values ± standard deviation (SD). Due to non-parametric data, a Kruskal–Wallis ANOVA was used with a confidence level of 95% with the STATISTICA 7.0 program.

## 4. Conclusions

Based on the results of this study, *A. polystachya* essential oil is an accessible and natural source of carvone. Furthermore, given the potent and promising anti-proliferative activity of the essential oil, novel anti-cancer formulations should be explored. In addition, due to its antioxidant power and the high percentage of carvone added to the presence of limonene and a series of compounds of terpenic origin present in *A. polystachya* essential oil, it can potentially be used as a food or drug preservative.

## Figures and Tables

**Table 1:** Essential oil composition of *A. polystachya*.

Nº	RT (min)	Components	% Area ^a^	RI ^b^	RI ^c^	Identification
1	11.08	*R*-limonene	4.10	1030	1031	RL, MS, Co
2	13.14	linalool	0.58	1105	1107	RL, MS, Co
3	13.77	*trans*-*p*-mentha-2,8-dienol	0.48	1112	1113	RL, MS
4	15.82	terpineol	0.45	1191	1189	RL, MS
5	15.98	dihydrocarvone	1.07	1200	1200	RL, MS, Co
6	17.31	*R*-carvone	91.03	1240	1240	RL, MS, Co
7	22.04	*E*-caryophyllene	0.92	1417	1418	RL, MS
8	23.78	α-curcumene	0.36	1481	1483	RL, MS
		Total identified	98.99			
		Hydrocarbon monoterpenes	4.10			
		Oxygenated monoterpenes	93.61			
		Hydrocarbon sesquiterpenes	1.28			

^a^ Surface area of GC peak; ^b^ RI: retention indices relative to C_8_–C_36_ *n*-alkanes on the HP-5 MS capillary column; ^c^ RI: retention index from the literature. RL: comparison of the RI with those of the literature [15]; MS: comparison of the mass spectra with those of the NIST 14; Co: co-elution with standard compounds available in our laboratory.

**Table 2:** Antioxidant activity of essential oil of the fresh leaves of *A. polystachya* and of three standards, determined by DPPH, H_2_O_2_, FRAP, and TRAP assays.

Antioxidant	IC_50_ (mg/mL)		FRAP (TEAC mM)	TRAP (TEAC mM)
	DPPH	H_2_O_2_		
Essential oil	38.34 ± 0.07 ^a^	15.67 ± 0.56 ^a^	29.44 ± 0.25 ^a^	1.01 ± 0.78 ^a^
*R*-Carvone	28.89 ± 0.03 ^b^	12.03 + 0.19 ^a^	30.05 ± 0.15 ^a^	1.02 ± 0.85 ^a^
*R*-limonene	20.84 ± 0.06 ^c^	1.08 ± 0.12 ^b^	0.46 ± 0.07 ^b^	0.42 ± 0.12 ^b^
Dihydrocarvone	55.31 ± 0.01 ^d^	25.89 ± 0.12 ^c^	14.34 ± 0.12 ^c^	0.84 ± 0.11 ^b^
BHT	n.d.	2.59 ± 0.05 ^d^	1.50 ± 0.04 ^d^	1.07 ± 0.45 ^a^
Trolox	0.11 ± 0.01 ^e^	2.85 ± 0.02 ^d^	n.d.	n.d.
Gallic acid	0.05 ± 0.12 ^e^	n.d.	1.80 ± 0.03 ^d^	1.11 ± 0.05 ^a^

Different letters in the same column indicate significant differences; *p* < 0.05, *n* = 3, n.d.: not detected.

**Table 3:** IC_50_^a^ (µg/mL) and selectivity index (SI) of *A. polystachya* essential oil and main components tested on MCF-7, PC-3, and HT-29 cancer cell lines, and on CCD 841 CoN normal cell line.

Samples	Cell Line			
	MCF-7	PC-3	HT-29	CCD 841 CoN
Essential oil	9.53 ± 0.45 *	6.74 ± 0.03 *	5.85 ± 0.39 *	27.81 ± 0.21 *
SI	2.92	4.13	4.75	
*R*-Carvone	>100	>100	>100	>100
*R*-limonene	>100	>100	>100	
Dihydrocarvone	>100	>100	>100	>100
Daunorubicin	0.33 ± 0.02 **	0.41 ± 0.04 **	15.11 ± 0.5 **	13.90 ± 0.3 **
SI	42.12	33.9	0.92	
5-FU	22.30 ± 0.2 ***	16.42 ± 0.6 ***	8.90 ± 0.7 ***	41.71 ± 0.3 ***
SI	1.87	2.54	4.69	

Different symbols in the same file indicate significant differences; *p* < 0.05, *n* = 3; ^a^ IC_50_ was evaluated using the sulforhodamine B (SRB) assay and ± is the standard deviation from three independent experiments.

## Data Availability

All data are available for scientific community.

## Supplementary Materials

The following are available online, Figure S1. GC–MS chromatogram of the essential oil of *Aloysia polystachya* from Chile.

## Sample Availability

Samples of the essential oil and compounds are available from the authors.
