# Supplementary material for: Antioxidant and Anti-Proliferative Activity of Essential Oil and Main Components from Leaves of Aloysia polystachya Harvested in Central Chile
Source: Molecules. 2020 Dec 30;26(1):131. doi: 10.3390/molecules26010131 (PMC7795351; doi:10.3390/molecules26010131)
Supplement: Supplementary file 1 [file molecules-26-00131-s001.pdf]

Supporting Information

Antioxidant and anti-proliferative activity of essential oil and Main Components from leaves of *Aloysia polystachya* harvested in central Chile

Alejandra Catalina Moller [1], Carol Parra [2], Bastian Said [3], Enrique Werner [4], Susana Flores [5], Joan Villena [6], Alessandra Russo [7], Nelson Caro [8], Iván Montenegro [9,*] and Alejandro Madrid [5,*]

[1] Escuela de Tecnología Médica, Facultad de Medicina, Universidad de Valparaíso, Angamos 655, Reñaca, Viña del Mar 2520000, Chile; alejandra.moller@uv.cl
[2] Laboratorio de Investigación en Nutrición y Alimentos (LINA), Departamento Disciplinario de Nutrición, Facultad de Ciencias de la Salud, Universidad de Playa Ancha, Valparaíso, CP 2340000, Chile; carol.parra@upla.cl
[3] Departamento de Química, Universidad Técnica Federico Santa María, Av. Santa María 6400, Vitacura 7630000, Santiago, Chile; bastian.said@usm.cl
[4] Departamento de Ciencias Básicas, Campus Fernando May, Universidad del Bío-Bío. Avda. Andrés Bello 720, casilla 447, Chillán 3780000, Chile; ewerner@ubiobio.cl
[5] Laboratorio de Productos Naturales y Síntesis Orgánica (LPNSO), Departamento de Química, Facultad de Ciencias Naturales y Exactas, Universidad de Playa Ancha, Avda. Leopoldo Carvallo 270, Playa Ancha, Valparaíso 2340000, Chile; E-Mail: s.flores.gonzalez@gmail.com; alejandro.madrid@upla.cl
[6] Centro de Investigaciones Biomedicas (CIB), Facultad de Medicina, Campus de la Salud, Universidad de Valparaíso, Angamos 655, Reñaca, Viña del Mar 2520000, Chile; E-Mail: juan.villena@uv.cl
[7] Department of Drug Sciences, University of Catania, Via S. Sofia 64, 95125 Catania, Italy; alrusso@unict.it
[8] Centro de Investigación Australbiotech, Universidad Santo Tomás, Avda. Ejército 146, Santiago 8320000, Chile; ncaro@australbiotech.cl
[9] Escuela de Obstetricia y Puericultura, Facultad de medicina, Universidad de Valparaíso, Angamos 655, Reñaca, Viña del Mar 2520000, Chile; ivan.montenegro@uv.cl

* Correspondence: ivan.montenegro@uv.cl and alejandro.madrid@upla.cl; Tel.: +56-032-250-0526 (A.M.)

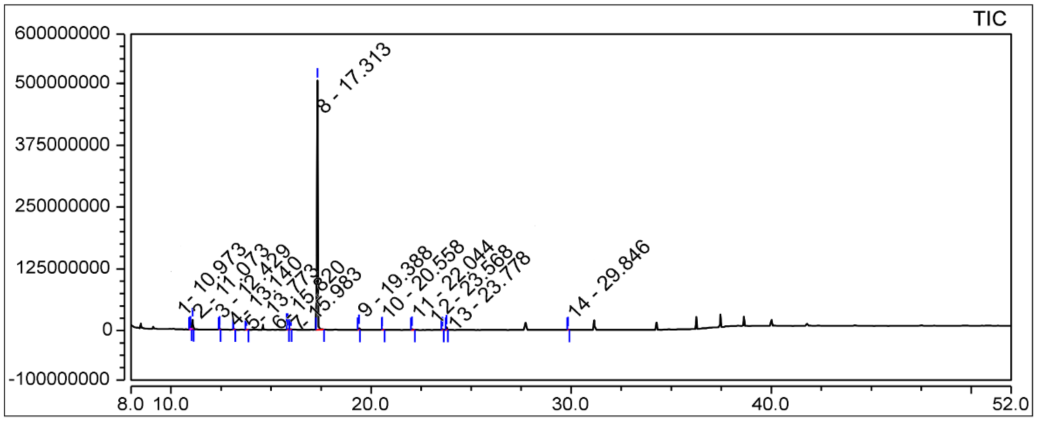

Figure S1. GC-MS chromatogram of the essential oil of *Aloysia polystachya* from Chile.
